# Translation, cultural adaptation and psychometric properties of the Ghanaian language (Akan; Asante Twi) version of the Health Literacy Questionnaire

**DOI:** 10.1186/s12913-020-05932-w

**Published:** 2020-11-23

**Authors:** Millicent Addai Boateng, Peter Agyei-Baffour, Sanne Angel, Ulrika Enemark

**Affiliations:** 1 Institute of Public Health, Aarhus University, Aarhus, Denmark; 2 Department of Health Policy Management and Economics; School of Public Health, Kwame Nkrumah University of Science and Technology, Kumasi, Ghana

**Keywords:** Health Literacy Questionnaire, Translation, Psychometric properties, Caregivers, Ghana, West Africa

## Abstract

**Background:**

Patients’ competencies and resources to manage their own health, which is termed health literacy, is a necessity for better health outcomes. Thus, it is relevant to have a comprehensive health literacy measurement tool suitable for populations of interest. The Health Literacy Questionnaire (HLQ) is a tool useful for health literacy assessment covering nine dimensions/scales of health literacy. The HLQ has been translated and validated in diverse contexts but has so far not been assessed in any country in sub-Saharan Africa. We sought to translate this tool into the most common language used in Ghana and assess its validity.

**Methods:**

We carried out a cross-sectional study using the HLQ concurrently with an assessment of a malaria programme for caregivers with children under 5 years. The HLQ was translated using a systematic translation procedure. We analysed the psychometric properties of the HLQ based on data collected by face-to-face interview of 1234 caregivers. The analysis covered tests on difficulty level of scales, composite reliability, Cronbach’s alpha and confirmatory factor analysis (CFA).

**Results:**

Cognitive testing showed that some words were ambiguous, which led to minor rewording of the questionnaire. A nine-factor CFA model was fitted to the 44 question items with no cross-loadings or correlated residuals allowed. Given the very restricted nature of the model, the fit was quite satisfactory: χ2 DWLS (866 df) = 17,177.58, *p* < 0.000, CFI = 0.971, TLI = 0.969, RMSEA = 0.126 and SRMR = 0.107. Composite reliability and Cronbach’s alpha were > 0.65 for all scales except Cronbach’s alpha for scale 9, ‘Understanding health information well enough to know what to do’ (0.57). The mean differences between most demographic groups among health literacy scales were statistically significant.

**Conclusion:**

The Akan-Twi version of HLQ proved relevant in our description of the health literacy levels among the caregivers in our study. This validated tool will be useful to conduct health literacy needs assessments to guide policies addressing such needs. Further work is needed to validate this tool for use in Ghana and similar contexts.

## Background

As health care is gradually becoming more patient-centred, patients may be expected to assume more responsibility for their own care process even in low resource health systems [1]. For patients to take on this responsibility, it is important to have the necessary competencies to make well-informed decisions [1, 2]. An individual’s ability to be actively involved in shared decision making together with health care providers improves self-management of illness and adherence to treatment [3, 4]. However, it is a health decision paradox that the increasing requirements to the individual to make proper health decisions are not always met by the appropriate support to help make these decisions [2]. Different factors influence the ability of individuals to understand the health information, follow health instructions and guidance, and ultimately make effective decisions related to their care and health [5]. Known factors include education and socio-economic status as well as health literacy, which receives considerable research attention [5].

The scope of health literacy has widened from ability to read and write health information to cover health promotion perspective and competencies needed to understand and apply health information while navigating complex health systems [6]. The World Health Organization (WHO) defines health literacy as ‘the cognitive and social skills which determine the motivation and ability of individuals to gain access to, understand and use information in ways which promote and maintain good health’ [2]. This definition highlights the multi-dimensional nature of the concept.

Nutbeam classifies health literacy into three levels; functional, interactive and critical health literacy [7]. He describes functional health literacy as the traditional means of health promotion and education where individuals are provided with information on health issues [7]. Interactive health literacy develops the skills of individuals in addition to providing health information. The third level, critical health literacy, promotes understanding of social, political and environmental determinants of health and improves community empowerment to act on these factors of health [7]. Differences in health literacy have been associated with observed health inequities among people of different race and educational levels [8]. In addition, since health literacy is associated with the ability to read and understand health information, the language used in healthcare delivery could play a significant role. This is very relevant in countries with diverse population backgrounds in terms of language and ethnicity [9, 10] as the official language may not be the language actually used.

Measurement of strengths and limitations of health literacy allows strategic design and delivery of interventions to address health inequities, improve health outcomes and strengthen health systems [11]. Thus, to select a measurement tool, it is necessary to consider the ability of the tool to describe the health literacy needs of people in certain social and health systems to provide the appropriate support. In recent years, the Health Literacy Questionnaire (HLQ) has been used to make a comprehensive measurement of health literacy [8].

The HLQ has been translated from English into several languages and the validated versions include German [12], Slovac [13], Danish [14], French [15], Norwegian [16] and Chinese [17]. The published validation studies [12–15, 17] show that the HLQ appears to be a robust tool to assess health literacy in different populations. The published reports on validation of the HLQ are performed in high or higher middle-income contexts. Despite the detailed scale components of the HLQ, making it user friendly for low and middle-income countries, there are no known validation studies on any African language. Thus, no reports on the validation of the HLQ in a West African or sub-Saharan African cultural context have been published.

As part of an assessment of the impact of a community malaria programme (Integrated Community Case Management of Malaria) in Ghana, we wanted to use the HLQ to assess the health literacy levels of caregivers with children under 5 years (target group of community programme). Ghana is a West African country with more than eighty (80) ethnic groups with their own language [18]. Akan is most widely spoken with three recognized dialects (Asante Twi, Fante and Akuapem Twi), of which Asante Twi is the most common dialect used by almost 100% in the study area. Asante -Twi is taught as one of the languages at basic education level in schools located in the southern part of Ghana and further taught at secondary and tertiary level as an area of specialty in languages. However, with English as the official language, institutions including the health system have health information written in English, with services often orally delivered in the local language. This may be because a greater percentage of the population use their mother-tongue in daily life and another reason could be because the cultural setting is one described to rely mostly on oral medium of communication [19]. The possibility of orally administering the HLQ [8] makes it more useful for settings like Ghana. This study reports on the translation, cultural adaptation and psychometric properties of the HLQ in the most common Ghanaian language, Akan, Asante-Twi.

## Methods

### Study design

We assessed the HLQ concurrently with an assessment of a malaria programme for caregivers with children under 5 years. We carried out a cross sectional survey from November to December 2017 in Ejisu-Juaben and Kwabre East, two peri-urban, predominantly farming municipalities in the Ashanti region of Ghana.

### Sample

We sampled 1270 caregivers with children under 5 years from the two selected municipalities. Two sub-municipal areas were randomly selected from each municipality and in each sub-municipal area; nine (9) communities were randomly selected. Subsequently, one community in Kwabre was excluded because it was very hard to reach due to logistics. Within each community, every other household was selected if there was a caregiver with a child under 5 years. However, if there was no caregiver with a child under 5 years, the interviewer continued to the next household until the required number of participants was reached. In households with more than one caregiver, one was selected based on caregiver consent and in difficult situations, randomly by tossing a coin.

### Data collection tool

The HLQ is a multi-dimensional tool developed to provide practitioners, organisations and governments with data on the health literacy strengths and limitations of individuals and populations [8]. The tool has been used to assess the needs and challenges of a wide range of people and organisations in various settings and is known for its excellent psychometric properties, construct validity and strong reliability with an unbiased mean estimate of group differences [11]. The HLQ is useful in surveys, intervention studies, and in uncovering the needs and capabilities of individuals [8]. Interpretations of HLQ data support decisions on changes in clinical treatments to suit the health literacy needs of patients [3, 20–23], develop group or population health literacy interventions [24] and to assess whether an intervention was successful in promoting health literacy of individuals or groups [25]. The HLQ covers nine conceptually distinct areas (scales) of health literacy including:

1. Feeling understood and supported by healthcare providers
2. Having sufficient information to manage health
3. Actively managing my health
4. Social support for health
5. Appraisal of health information
6. Ability to actively engage with healthcare providers
7. Navigating the healthcare system
8. Ability to find good health information
9. Understand health information well enough to know what to do.

The scales are based on 44 question items (Table 2). The question items of the first part covering scales 1–5 are scored on a 4-point Likert scale (Strongly disagree, Disagree, Agree, Strongly agree), while a 5-point Likert scale is used for the second part covering scales 6–9, which rates the ability to perform various tasks (cannot do, very difficult, difficult, easy and very easy).

The questionnaire provides low and high descriptors for each of the nine scales, which explains the scope of the elements of the scales in relation to health literacy and in addition, it provides the intent of each item within each scale [8]. These descriptions and intent guided the translation process as they outline the intended meaning and conceptual basis for the items [8] Validation is important in the translation of questionnaires to other languages, to ensure the questions are correctly asked as intended. Although construct validity is estimated from statistical imputations post translation, it is a process that should include evidence to support the quality and credibility of inferences made from the scale scores [25]. Therefore, it is important that the translation process is validity driven, especially in this study, given the cultural and linguistic differences between the context of the developers of the original version of the questionnaire [8] and this study’s context.

#### Translation process

The tool was translated following the Translation Integrity Procedure (TIP) [8]. The Translation Integrity Procedure is a systematic process of translation using a translation management grid and item intent format [8] developed by two of the developers of the HLQ [25]. The item intent format includes high and low definitions of the HLQ constructs and describes the intended meaning of each item. It further provides an in-depth explanation about the intent and conceptual basis of the items and spells out synonyms for words and phrases in each item. The translation management grid and item intent format serve as the primary support and guidance for translators and the key focus for the team consensus discussion of the translation. The process included forward translation, backward translation, and translation consensus discussions which served as a group cognitive discussion.

#### Forward translation

Two forward translators from the Department of Languages at the Kwame Nkrumah University of Science and Technology translated the original English versions of the HLQ into Asante Twi. These translators were selected based on their rich expertise in the local language as well as their experience in translation to and from the local language. In line with the TIP, the translators each provided individual versions and consensus on an appropriate translation version was reached following discussion.

This study carried out individual interviews on the provisional forward translation in a similar setting as our study site to evaluate how well the translated questionnaire was understood at community level; this resulted in minor adaptations. Observations showed that participants found it difficult to respond to some of the questions due to the phrasing and the Twi vocabulary used in some of the questions and sentences. Suggestions from the participants interviewed were recorded and the forward translators revised their first version based on the report from the individual interviews.

#### Backward translation

Unlike the other translated versions of the HLQ using backward translation by native English speakers speaking the local language of interest, this study used a slightly different approach. Although the Ghanaian language selected for the translation is the most common language in Ghana, it does not have any international recognition relative to the other languages [12–15, 17] with translated versions of the HLQ. Thus, it was a challenge to find a native English speaker who was competent in the Asante Twi language. Thus, we resorted to the use of indigenous experts in the field of translation in the backward translation. Thus, the backward translator was selected based on translation experience. To avoid any bias, the person was from the Department of Languages at another institution (Wesley College). This person was very resourceful in the English language as well as a good command of the local language. The translator was blinded to the original English version of the questionnaire and was asked to translate the finalised version of the forward translators back into English.

#### Translation consensus discussion

A consensus meeting was organised for all the translators together with the project team to compare the forward and backward translations against the original English versions and the item intent form of the HLQ. Relevant changes were made to arrive at the same meaning as the original version. In most cases, the forward translations were accepted, and the backward version revised. In the local language, one word may be used for more than one English word, but the meaning depends on the context in which it is used. For example, one word or phrase in the local language could cover ‘accurately follow’ and ‘adhere to’. Therefore, in one question, the backward translation replaced ‘accurately follow’ with ‘adhere to’ based on the forward translation. Here, the translation was discussed and revised accordingly in the backward translation without changing the forward translation. For some other questions, revision was necessary due to an exaggeration of the intended meaning. For example, the word ‘understand’ which was translated as ‘perfectly understand’ was considered to depict a stronger meaning than the original version. Similarly, the consensus panel accepted the forward translations (local version) because it fitted the intended meaning of the original version. The consensus meeting between the Ghana translation team and the head of the HLQ team in Australia was held as an online Skype meeting. The tool was finalised after this consensus meeting and prepared for fieldwork testing.

### Data collection

Eight research assistants collected data through house visits. The HLQ was administered orally alongside a questionnaire on malaria. Interviews lasted 20–30 min. The questionnaire data were entered into an open data kit software allowing the interviewer to enter data directly into the dataset. Data were collected for 1234 participants, as data from 36 participants were lost during the data synchronisation process. Participants were given cereals (worth less than $1.00) for their children after the interviews to spur the interest in participation in the study.

### Data analysis

The data analysis included test on difficulty level of scales, composite reliability, Cronbach’s alpha and confirmatory factor analysis. We used the R statistical software for the confirmatory factor analysis and otherwise STATA version 15.

Each of the 44 question items was described based on their mean score, median, total and percentage score in each of the response categories as well as the difficulty level across the nine scales. The HLQ was developed to ensure that items in each scale were sensitive to cover the full spectrum of health literacy capabilities ranging from mild, moderate or severe limitations [8]. Thus, the scales were developed to cover a range of item difficulty levels, where a more difficult item is one which fewer people would give a maximum score (strongly agree or find very easy) [8]. Difficulty level of scales was calculated as the proportion of responses on ‘disagree’ or ‘strongly disagree’ to responses on ‘agree’ or ‘strongly agree’ for scales 1–5, and as the proportion of responses on ‘cannot do’, ‘very difficult’ or ‘quite difficult’ against ‘quite easy’ and ‘very easy’ for scales 6–9 [8]. The difficulty level of a scale showed its sensitivity towards people with mild, moderate or severe health literacy limitations. Thus, ability to score low or high on the scale should reflect an individual’s challenges and strengths in health literacy.

Since HLQ scales were stated a priori, we used confirmatory factor analysis (CFA) to test factor structure. The recommended estimation procedure for running the CFA for ordinal variables is the Diagonal Weighted Least Squares (DWLS) [26], which is not available in Stata. Mplus was considered as in other validation studies, but this study opted for R for cost reasons. The LAVAAN (Latent variable analysis) package, available on R, is used to estimate multivariate statistical models including CFA using DWLS [27]. The numerical findings from the LAVAAN package are noted to be similar to that of the Mplus software programme [27].

For the CFA, we first fitted a model to the data for each of the confirmed scales. The one-factor CFA model analysis provided the standardized and unstandardized factor loadings of the observed variables to their latent variables together with R^2^ (the variance in the observed variable explained by the latent variable), standard errors, 95% confidence intervals and variance. The result of the analysis also included the various model fit indexes; Chi-square, Comparative Fit Index (CFI), Tucker Lewis Index (TLI), Root Mean Square Error of Approximation (RMSEA) and the Standard Root Mean Square Residual (SRMR). In line with the original HLQ validation study [8], we report on the indexes with the following threshold values for the test of good fit; CFI > 0.95, TLI > 0.95, SRMR< 0.08 and RMSEA< 0.06, though a value of RMSEA< 0.08 was set as a reasonable fit. Then, a full nine-factor CFA model with no correlated residuals or cross-loadings was fitted to the data to investigate discriminant validity. As in other translated versions of HLQ [13–15, 17], we estimated the composite reliability in addition to Cronbach’s alpha with the knowledge that α is a biased estimate of population reliability. In addition, this estimate helps for comparison with other HLQ translation validation studies.

Finally, analysis was carried out using a one-way ANOVA test to assess the mean differences on the HLQ scale across a range of socio-demographic groups. We report on the effect size, with 95% confidence intervals for differences in mean between the groups and this was calculated using Cohen d’ with interpretation of effect size as: “small” ES < 0.20 to 0.50; “medium” ES is between 0.50 and 0.80 and “large” ES > 0.80.

## Results

### Cognitive interviews

The cognitive interviews based on the translated questionnaire resulted in a few changes in the forward translation version of the questionnaire. The main challenge was how to phrase the questions in the second part of the questionnaire. Literally, reading out the statements in the second part e.g. Item 7.1 ‘Find the right healthcare’ in the local language sounded commanding, thus they were rephrased into questions. Therefore, the research assistants had to be conscious of this approach to avoid confrontations with respondents. “Words like healthcare, and health were quite difficult to translate because they are normally referred to by the same word in the study context “apomuden”. However, the translated word was accepted in the forward translation because the it did not change the intended meaning. Similarly, ‘healthcare provider’ was translated as health worker since healthcare provider is often referred to the health facilities in the context. In the item 5.2, “I have at least one healthcare provider who knows me well” the phrase ‘knows me well’ was translated as “onim me paa” which literally translates in English as knows me very well. This was projected to be stronger than the intended meaning, however, in the study context, well and very well are often used synonymously although ‘knows me very well’ could be translated as ‘onim me yie’ which is much stronger. If the phrase used was ‘knows me’, then the word ‘well’ would not have been added which would make it less strong. Since the word well was needed in the item, we kept the forward translation. Overall, only few words were changed with minor editing at the consensus meeting with the developers of the questionnaire.

### Sample background characteristics

Table 1 describes the demographic characteristics and self-reported illness or long-term disability status of respondents. 98% of respondents were females and respondents reported that 65% of household heads were men. With a mean age of respondents of approximately 31 years, the most represented age group consisted of respondents within the ages 25–44. About 72% of respondents had nine or less years of schooling and 2% had more than 12 years of schooling. The majority of respondents (54%) were employed with few retirees (5%). Almost 25% of respondents reported to be living alone and in terms of self-reported illness or long-term disability, most of the respondents (62%) reported no illness or long-term disability; 10% of the respondents reported to have more than one illness or long-term disability.

**Table 1:** Demographic, socio-economic and health characteristics of respondents (*N* = 1234)

Background characteristics	Frequency	Percentage
Gender		
Female	1204	97.6
Male	30	3.4
Sex of Head of household		
Male	808	65.5
Female	426	34.5
Age groups^a^		
15–24	238	19.4
25–44	945	76.9
45–69	46	3.7
Years of schooling		
≤ 9 years	895	72.5
> 9 ≤ 12 years	309	25.1
> 12 years	30	2.4
Employment		
Employed	673	54.5
Unemployed	499	40.5
Retired	62	5.0
Living alone		
No	927	75.1
Yes	307	24.9
Self-reported illness or long-term disability		
At least one chronic or long-term illness^b^	115	9.3
Only non-chronic illness	354	28.7
None	765	62.0

^a^*N* = 1229

^b^Of the 9% who self-reported chronic illness, the most prevalent was heart problems (38%), asthma (16%), depression (12%) and diabetes (13%). Others included hypertension, stroke, back pain, cancer and arthritis

### Difficulty level

Table 2 shows the difficulty levels of the various items of the HLQ as well as the average difficulty level for each of the nine scales. Below we report on scales and items with highest and lowest difficulty levels.

**Table 2:** Health Literacy Questionnaire with mean scores and difficulty levels for scales and items

**Table 2A – PART 1 of HLQ**								
**Questions (Part 1)**	**Median**	**Mean (SD)**	**Strongly Disagree (%)**	**Disagree (%)**	**Agree (%)**	**Strongly agree (%)**		**Difficulty Level (%) (CI)**
**Scale 1: Feeling Understood and Supported by Healthcare Providers**								
1. I have at least one healthcare provider … … .	2	2.39 (0.78)	12.7	41.1	40.1	6.1		53.8 (51.0–56.6)
2. I have at least one healthcare provider I can discuss … …	2	2.40 (0.74)	10.4	44.2	40.5	4.9		54.5 (51.7–57.3)
3. I have the healthcare providers I need … … .	2	2.46 (0.66)	6.3	44.1	46.3	3.3		50.4 (47.6–53.2)
4. I can rely on at least one healthcare provider	2	2.50 (0.73)	6.4	44.9	41.1	7.5		51.4 (48.6–54.2)
**Average for Scale 1**		**2.44 (0.57)**						**52.5**
**Scale 2: Having Sufficient Information to Manage My Health**								
1. I feel I have good information … … .	3	2.81 (0.55)	2.2	19.8	72.4	5.7		21.9 (19.7–24.4)
2. I have enough information to help me deal … .	3	2.59 (0.58)	3.3	36.0	59.0	1.7		39.3 (36.6–42.1)
3. I am sure I have all the information I need to manage … .	3	2.55 (0.59)	2.9	41.2	53.9	1.9		44.2 (41.4–46.9)
4. I have all the information I need to look after … .	3	2.52 (0.59)	3.5	42.1	53.1	1.4		45.5 (42.8–48.3)
**Average for Scale 2**		**2.62 (0.41)**						**37.7**
**Scale 3: Actively Managing My Health**								
1. I spend quite a lot of time actively … .	3	2.62 (0.63)	4.7	32.1	59.8	3.4		36.8 (34.1–39.5)
2. I make plans for what I need to … .	3	2.63 (0.62)	4.3	31.6	60.9	3.2		35.9 (33.3–38.6)
3. Despite other things in my life, I make time … ..	3	2.64 (0.60)	3.6	32.0	61.5	2.9		35.6 (32.9–38.3)
4. I set my own goals about health … .	3	2.78 (0.60)	3.5	20.9	70.0	5.6		24.4 (22.1–26.9)
5. There are things that I do regularly to make … .	3	2.81 (0.52)	1.8	19.9	74.1	4.1		21.7 (19.5–24.1)
**Average for Scale 3**		**2.69 (0.42)**						**30.9**
**Scale 4: Social Support for Health**								
1. I can get access to several people who … .	3	2.63 (0.62)	3.6	33.8	58.5	4.0		37.4 (34.8–40.2)
2. When I feel ill, the people around me … .	3	2.74 (0.64)	4.5	23.1	66.0	6.4		27.6 (25.2–30.2)
3. If I need help, I have plenty of people I … .	3	2.58 (0.66)	4.7	38.2	51.9	5.2		42.9 (40.1–45.6)
4. I have at least one person who can … .	3	2.70 (0.67)	4.4	29.2	58.8	7.6		33.5 (30.9–36.2)
5. I have strong support from my family … .	3	2.64 (0.66)	4.4	33.0	56.5	6.2		37.3 (34.7–40.1)
**Average for Scale 4**		**2.66 (0.43)**						**35.7**
**Scale 5: Appraisal of Health Information**								
1. I compare health information from diff … .	3	2.64 (0.58)	4.3	29.0	65.4	1.3		33.3 (30.7–35.9)
2. When I see new information about health, … .	3	2.46 (0.67)	8.5	38.0	52.2	1.3		46.5 (43.7–49.3)
3. I always compare health information from different sources	3	2.63 (0.58)	3.9	30.6	64.0	1.5		34.5 (31.9–37.2)
4. I know how to find out if the health information I receive … .	3	2.67 (0.59)	2.8	30.3	63.2	3.6		33.1 (30.6–35.8)
5. I ask healthcare providers about the quality … .	2	2.45 (0.65)	6.9	43.2	48.0	1.9		50.1 (47.2–52.9)
**Average for Scale 5**		**2.57 (0.43)**						**39.5**
**Table 2B- PART 2 OF HLQ**								
			**Cannot do/always difficult %**	**Usually difficult%**	**Sometimes difficult %**	**Usually Easy %**	**Always Easy %**	**DL GH**
**Scale 6: Ability to Actively Engage with Healthcare Providers**								
1. Make sure that healthcare providers understand your …	4	3.87 (0.78)	0.6	6.2	15.6	60.4	17.2	22.4 (20.1–24.8)
2. Feel able to discuss your health concerns with …	4	3.30 (0.89)	2.5	18.6	28.4	47.4	3.1	49.5 (46.7–52.3)
3. Have good discussions about your health with doctors	4	3.30 (0.90)	2.8	18.0	28.8	47.1	3.3	49.5 (46.8–52.4)
4. Discuss things with healthcare providers until … .	4	3.28 (0.90)	3.4	18.5	26.6	49.4	2.1	48.5 (45.7–51.2)
5. Ask healthcare providers questions to get the …	4	3.37 (0.86)	2.3	14.7	30.1	49.0	3.8	47.2 (44.4–49.9)
**Average for Scale 6**		**3.43 (0.64)**						**43.4**
**Scale 7: Navigating the Healthcare System**								
1. Find the right healthcare	4	3.30 (0.89)	2.9	17.8	27.8	49.3	2.2	48.5 (45.7–51.3)
2. Get to see the healthcare provider I need to	4	3.39 (0.89)	1.5	18.1	25.4	49.7	5.4	44.9 (42.1–47.7)
3. Decide which healthcare provider you need to see	3	3.18 (0.95)	4.0	23.8	23.6	46.7	1.9	51.4 (48.7–54.2)
4. Make sure you find the right place to get the health …	4	3.53 (0.77)	1.4	10.7	23.6	61.9	2.4	35.6 (33.0–38.4)
5. Find out what healthcare services you are entitled … .	3	3.28 (0.85)	2.3	17.6	31.1	47.2	1.7	51.0 (48.7–54.3)
6. Work out what is the best care for you	4	3.31 (0.86)	3.2	15.5	30.1	50.0	1.2	48.8 (45.9–51.5)
**Average for Scale 7**		**3.33 (0.63)**						**46.7**
**Scale 8: Ability to Find Good Health Information**								
1. Find information about health problems	4	3.50 (0.86)	1.9	11.0	30.1	49.3	7.7	43.0 (40.3–45.8)
2. Find health information from several different places	4	3.62 (0.73)	1.1	7.9	22.8	64.8	3.4	31.8 (29.2–34.4)
3. Get information about health so you are up to date	4	3.42 (0.75)	1.0	10.8	35.7	50.4	2.1	47.5 (44.7–50.3)
4. Get health information in words you …	4	3.84 (0.82)	2.8	3.6	15.5	63.5	14.7	21.8 (19.6–24.2)
5. Get information by yourself	3	2.77 (1.12)	18.4	21.1	26.6	33.0	0.9	66.1 (63.4–68.7)
**Average for Scale 8**		**3.36 (0.58)**						**42.0**
**Scale 9: Understanding Health Information Well Enough to Know What to Do**								
1. Confidently fill in medical forms in the … .	4	3.49 (1.01)	7.8	7.0	22.0	54.5	8.7	36.8(34.1–39.5)
2. Accurately follow the instructions from … .	4	3.89 (0.72)	–	3.9	20.4	58.4	17.3	24.3(21.9–26.8)
3. Read and understand written health … .	3	2.69 (1.17)	21.3	22.2	24.9	29.0	2.6	68.4(65.7–70.9)
4. Read and understand all the information on … .	3	2.78 (1.21)	21.6	18.1	24.5	32.0	3.7	64.3(61.5–66.9)
5. Understand what health providers … .	4	3.91 (0.73)	0.2	2.2	19.0	63.8	14.91	21.3(19.1–23.7)
**Average for Scale 9**		**3.35 (0.60)**						**43.02**

For scales 1–5, scale 3 ‘Actively managing my health’ showed the lowest difficulty level with an item average difficulty of 0.31. Scale 1, ‘Feeling understood and supported by healthcare provider’ recorded the highest difficulty level with an item average difficulty of 0.52. Thus, on average respondents easily scored high (agreed) on scale 3 and found it hard to score high on scale 1.

For scales 6–9, scale 8 ‘Ability to find good health information’ had the lowest difficulty level, with an item average difficulty of 0.42. Scale 7, ‘Navigating the healthcare system’ showed the highest difficulty level, with an item average difficulty of 0.47. Thus, respondents found it easier to score high on scale 8 than scale 7.

At the item level for scales 1–5, two items had the same difficulty level of 0.22, which was the lowest difficulty level among the items under scales 1–5. The two items included item 2.1 ‘I feel I have good information about health’ and item 3.5, ‘There are things I do regularly to make myself healthier’. The item that showed the highest difficulty level in part 1 was found in scale 1, item 1.1, ‘I have at least one healthcare provider who knows me well’ with a difficulty level of 0.54.

For scales 6–9, the item with highest difficulty level was in scale 9, item 9.3, ‘Read and understand written health information’ with a difficulty level of 0.68. The item with least difficulty level was also in scale 9, item 9.5 ‘Understand what health providers are asking you to do’ with a difficulty level of 0.21.

Comparatively, the range of difficulty levels on average for scales 1–5 was relatively lower (range 0.22–0.55; mean: 0.33) than in scales 6–9 (0.21–0.68; 0.47). The scale with the lowest range for item difficulty level was scale 1 ‘Feeling understood and supported by healthcare providers’ (0.54–0.50; 0.04). The scale with the highest range for item difficulty level was scale 9 ‘Understanding health information well enough to know what to do’ (0.21–0.68; 0.47).

### Psychometric properties

Table 3 shows the psychometric measures and the findings from the one factor CFA. Generally, most of the items loaded well on the various scales but there were seven items across five scales with factor loadings of 0.4 or less. The seven items included:

- item 1 in scale 2, (I feel I have good information about health; 0.37);
- item 4 in scale 4, (I have at least one person who come to medical appointments with me; 0.40);
- item 1 in scale 6, (Make sure that healthcare providers understand your problems properly; 0.34);
- item 5 in scale 8, (Get health information by yourself; 0.23);
- item 1 in scale 9, (Confidently fill in medical forms in the correct way; 0.31);
- item 2 in scale 9, (Accurately follow the instructions from healthcare providers; − 0.13) and
- item 5 in scale 9, (Understanding what healthcare providers are asking you to do; − 0.07).

**Table 3:** Psychometric properties of the Health Literacy Questionnaire - Asante Twi

Item	Factor loading (95% CI)	R^2^	Composite reliability	Cronbach’s alpha
Scale 1: Feeling understood and supported by healthcare providers			0.80	0.80 (0.78–0.82)
I have at least one healthcare provider who knows me well	0.76 (0.73–0.78)	0.58		
I have at least one health care provider I can discuss my health with	0.86 (0.84–0.89)	0.74		
I have the healthcare providers I need to help me work out what I need to do	0.82 (0.80–0.84)	0.67		
I can rely on at least one healthcare provider	0.61 (0.58–0.64)	0.37		
Model fit: χ^2^_DWLS_ (2) = 20.643, ρ = 0.000, CFI = 0.998, TLI = 0.993, RMSEA = 0.087 (0.056–0.123)				
2. Having sufficient information to manage my health			0.69	0.68 (0.65–0.71)
I feel I have good information about health	0.37 (0.31–0.42)	0.14		
I have enough information to help me deal with my health problems	0.73 (0.69–0.76)	0.53		
I am sure I have all the information I need to manage my health effectively … .	0.74 (0.71–0.77)	0.55		
I have all the information I need to look after my health … .	0.79 (0.75–0.82)	0.62		
Model fit: χ^2^_DWLS_ (2) = 8.573, ρ = 0.014, CFI = 0.998, TLI = 0.994, RMSEA = 0.052 (0.020–0.089)				
3. Actively managing my health			0.75	0.75 (0.73–0.77)
I spend quite a lot of time actively managing my health … .	0.70 (0.66–0.74)	0.49		
I make plans for what I need to do to be healthy … ..	0.71 (0.68–0.75)	0.50		
Despite other things in my life, I make time to be healthy … .	0.74 (0.71–0.77)	0.55		
I set my own goals about health and fitness … .	0.65 (0.61–0.69)	0.42		
There are things that I do regularly to make myself more healthy	0.67 (0.63–0.71)	0.45		
Model fit: χ^2^_DWLS_ (5) = 179.252, ρ = 0.000, CFI = 0.965, TLI = 0.930, RMSEA = 0.168 (0.148–0.190)				
4. Social support for health			0.69	0.68 (0.65–0.71)
I can get access to several people who understand and support …	0.68 (0.64–0.72)	0.46		
When I feel ill, the people around me really understand what … ..	0.52 (0.47–0.56)	0.27		
If I need help, I have plenty of people I can rely … .	0.74 (0.71–0.78)	0.55		
I have at least one person who can come to medical appointments … ..	0.40 (0.35–0.5)	0.16		
I have strong support from family or friends … ..	0.72 (0.68–0.75)	0.52		
Model fit: χ^2^_DWLS_ (5) = 23.658, ρ = 0.000, CFI = 0.995, TLI = 0.989, RMSEA = 0.055 (0.034–0.078)				
5. Appraisal of health information			0.75	0.74 (0.72–0.77)
I compare health information from different sources … ..	0.80 (0.77–0.83)	0.64		
When I see new information about health, I check up … … .	0.72 (0.68–0.75)	0.52		
I always compare health information from different sources and decide … .	0.83 (0.80–0.85)	0.69		
I know how to find out if the health information I receive is right … .	0.49 (0.45–0.63)	0.24		
I ask healthcare providers about the quality of the health information … .	0.59 (0.54–0.63)	0.35		
Model fit: χ^2^_DWLS_ (5) = 74.552, ρ = 0.000, CFI = 0.987, TLI = 0.974, RMSEA = 0.106 (0.086–0.128)				
6. Ability to actively engage with health care providers			0.81	0.79 (0.78–0.81)
Make sure that healthcare providers understand your problems properly	0.34 (0.29–0.39)	0.11		
Feel able to discuss your health concerns with a healthcare provider	0.84 (0.81–0.86)	0.71		
Have good discussions about your health with doctors	0.80 (0.78–0.82)	0.64		
Discuss things with healthcare providers until you understand all you need to	0.82 (0.80–0.85)	0.67		
Ask healthcare providers questions to get the health information you need	0.75 (0.72–0.77)	0.56		
Model fit: χ^2^_DWLS_ (5) = 20.230, ρ = 0.001, CFI = 0.999, TLI = 0.997, RMSEA = 0.050 (0.028–0.073)				
7. Navigating health system			0.82	0.82 (0.81–0.84)
Find the right health care	0.66 (0.62–0.69)	0.44		
Get to see the healthcare providers you need to	0.70 (0.67–0.73)	0.49		
Decide which healthcare provider you need to see	0.77 (0.74–0.80)	0.59		
Make sure you find the right place to get the health care you need	0.65 (0.61–0.68)	0.42		
Find out which healthcare services you are entitled to	0.73 (0.69–0.76)	0.53		
Work out what the best care is for you	0.77 (0.74–0.79)	0.59		
Model fit: χ^2^_DWLS_ (9) = 17.352, ρ = 0.043, CFI = 0.999, TLI = 0.999, RMSEA = 0.027 (0.005–0.047)				
8. Finding health information			0.66	0.66 (0.63–0.69)
Find information about health problems	0.83 (0.80–0.86)	0.69		
Find health information from several different places	0.69 (0.65–0.72)	0.48		
Get information about health so you are up to date with the best … …	0.74 (0.71–0.78)	0.55		
Get health information in words you understand	0.60 (0.56–0.64)	0.36		
Get health information by yourself	0.23 (0.18–0.29)	0.05		
Model fit: χ^2^_DWLS_ (9) = 70.279, ρ = 0.000, CFI = 0.985, TLI = 0.970, RMSEA = 0.103 (0.082–0.125)				
9. Understanding health information well enough to … ..			0.71	0.57 (0.54–0.61)
Confidently fill in medical forms in the correct way	0.31 (0.27–0.35)	0.10		
Accurately follow the instructions from healthcare … .	−0.13 (− 0.19- (− 0.08))	0.02		
Read and understand written health information	0.90 (0.86–0.93)	0.81		
Read and understand all the information on medication …	0.98 (0.94–1.01)	0.96		
Understand what healthcare providers are asking you to do	−0.07 (− 0.12-(− 0.02))	0.00		
Model fit: χ^2^_DWLS_ (5) = 944.273, ρ = 0.000, CFI = 0.950, TLI = 0.901, RMSEA = 0.390 (0.370–0.412)				

The model fit for each scale was generally good with a close fit for scales 2, 4, 6 and 7 with a RMSEA≤0.05. The model fit for scale 9 (Understanding health information well enough to know what to do) did not perform well. The TLI was < 0.95 and RMSEA was high > 0.1 in both the point estimate and the confidence interval. Reliability coefficients (for both Cronbach’s alpha and composite reliability) ≥0.8 for three scales (1, 6, 7) and ≥ 0.7 for two scales (3, 5) were observed. Scales 2, 4 and 8 had reliability coefficient of 0.69, 0.69 and 0.66, respectively. For scale 9, the reliability coefficient from Cronbach’s alpha (0.57) seemed to differ from that of composite reliability (0.71). Since composite reliability is much more acceptable, this study relies on the latter.

A nine-factor CFA model was fitted to the 44 items with no cross-loadings or correlated residuals allowed. Given the very restricted nature of the model, the fit was quite satisfactory: χ^2^ DWLS (866 df) = 17,177.58, *p* < 0.000, CFI = 0.971, TLI = 0.969, RMSEA = 0.126 and SRMR = 0.107. It is not surprising that there are low estimates for both CFI and TLI with high estimates for SRMR because the model contains large parameters set precisely to 0.0. The ranges of the factor loadings in this model were scale 1) ‘Feeling understood and supported by healthcare providers’: 0.65–0.87; scale 2) ‘Having sufficient information to manage my health’: 0.43–0.75; scale 3) ‘Actively managing my health’: 0.63–0.77; scale 4) ‘Social support for health’: 0.52–0.72; scale 5) ‘Appraisal of health information’: 0.51–0.76; scale 6) ‘Ability to actively engage with healthcare providers’: 0.53–0.82; scale 7) ‘Navigating the healthcare system’: 0.69–0.78; scale 8) ‘Ability to find good health information’: 0.57–0.77; and scale 9) ‘Understanding health information well enough to know what to do’: 0.55–0.84. An inter-factor correlation showed positive correlation coefficients among scales with the strongest correlations between 6, 7 and 8 (6/7–0.72, 7/8–0.73). Scale 9 had the lowest correlation coefficients with all scales ranging from 0.17(9/1) to 0.50 (9/8).

### Health literacy profiles of the sample

Table 4 shows patterns of HLQ scores in relation to demographic and other characteristics. The mean difference in scores between gender groups for all scales was not statistically significant. However, with only 2% of respondents being male, there isn’t enough evidence to make any conclusions. The mean difference between the age groups was statistically significant for some scales including: scale 2) ‘Having sufficient health information’, scale 3) ‘Ability to manage health’, scale 4) ‘Social support for health’ and scale 9) ‘Understanding health information well enough to know what to do’. The mean difference between education groups was statistically significant for all scales. Thus, respondents with > 12 years of schooling had higher scale scores on the average than those with ≤12 years of schooling. The mean difference between language groups was statistically significant for all scales. Thus, respondents who spoke English performed significantly better statistically than those who spoke the local language.

**Table 4:** Patterns of mean HLQ scale scores in relation to demographics and other characteristics

Variable	1. Feeling understood and supported by healthcare providers	2. Having sufficient information to manage my health	3. Actively managing my health	4. Social support for health	5. Appraisal of health information	6. Ability to actively engage with healthcare providers	7. Navigating the healthcare system	8. Ability to find good health information	9. Understanding health information enough to know what to do
Sex									
Female	2.44(0.58)	2.62 (0.41)	2.69 (0.42)	2.66 (0.43)	2.57 (0.43)	3.43 (0.64)	3.33 (0.63)	3.36 (0.59)	3.35 (0.60)
Male	2.47 (0.51)	2.54 (0.39)	2.74 (0.55)	2.73 (0.39)	2.52 (0.46)	3.31 (0.57)	3.20 (0.67)	3.45 (0.46)	3.47 (0.63)
*P*-value	*p* = 0.3981	*p* = 0.1483	*p* = 0.2723	*p* = 0.1897	*p* = 0.2559	*p* = 0.1509	*p* = 0.1208	*p* = 0.1991	*p* = 0.1341
Effect size (95% CI)	0.05 (− 0.32 to 0.41)	− 0.20 (− 0.56 to 0.17)	0.11 (− 025 to 0.47)	0.16 (− 0.20 to 0.52)	− 0.12 (− 0.48 to 0.24)	−0.19 (− 0.55 to 0.17)	− 0.22 (− 0.58 to 0.14)	0.14 (− 0.22 to 0.50)	0.21 (− 0.16 to 0.57)
Age									
15–35	2.43 (0.57)	2.61(0.42)	2.68(0.43)	2.68(0.42)	2.57(0.44)	3.43(0.64)	3.34(0.64)	3.42(0.57)	3.41(0.59)
36–69	2.48 (0.59)	2.66(0.40)	2.74(0.40)	2.61(0.46)	2.58(0.43)	3.43(0.64)	3.31(0.62)	3.44(0.57)	3.21(0.59)
*P*-value	*P* = 0.0719	*P* = 0.0270	*P* = 0.0121	*P* = 0.0058	*P* = 0.2876	*P* = 0.4420	*P* = 0.2009	*P* = 0.3056	*p* < 0.001
Effect size (95% CI)	− 0.10 (− 0.22 to 0.03)	− 0.13 (− 0.25 to 0.00)	− 0.15 (− 0.28 to − 0.02)	0.17 (0.04 to 0.29)	− 0.04 (− 0.17 to 0.09)	−0.01 (− 0.14 to 0.12)	0.06 (− 0.07 to 0.18)	−0.03 (− 0.16 to 0.10)	0.33 (0.21 to 0.46)
Years of schooling									
≤ 12 years	2.43(0.57)	2.62(0.41)	2.70(0.42)	2.65(0.43)	2.60(0.43)	3.42(0.64)	3.33(0.63)	3.42(0.57)	3.34(0.59)
> 12 years	2.65(0.67)	2.75(0.47)	2.87(0.54)	2.83(0.51)	2.75(0.53)	3.66(0.73)	3.64(0.67)	3.69(0.55)	4.01(0.57)
*P*-value	*P* = 0.0215	*P* = 0.0401	*p* = 0.0090	*p* = 0.0123	*P* = 0.0099	*P* = 0.0219	*P* = 0.0038	*P* = 0.0048	*p* < 0.001
Effect size (95% CI)	−0.37 (−0.74 to −0.01)	−0.32 (− 0.70 to 0.04)	−0.44 (− 0.80 to − 0.07)	−0.42 (− 0.78 to − 0.05)	−0.43 (− 079 to − 0.07)	−0.37 (− 0.73 to − 0.01)	−0.49 (− 0.86 to − 0.13)	−0.48 (− 0.84 to − 012)	− 1.15 (−1.51 to − 0.78)
Language									
Local	2.42(0.58)	2.60(0.41)	2.67(0.42)	2.64 (0.43)	2.55(0.43)	3.41(0.63)	3.31(0.63)	3.34(0.58)	3.30(0.58)
English	2.58(0.55)	2.78(0.40)	2.84(0.39)	2.81(0.38)	2.76(0.40)	3.58(0.68)	3.50(0.65)	3.58(0.57)	3.81(0.56)
*P*-value	*p* = 0.0016	*p* < 0.001	*p* < 0.001	*p* < 0.001	*p* < 0.001	*p* = 0.0016	*p* = 0.0011	*p* < 0.001	*p* < 0.001
Effect size (95% CI)	−0.27 (−0.45 to −0.09)	−0.44 (− 0.62 to − 0.26)	−0.41 (− 0.59 to − 0.23)	−0.40 (− 0.58 to − 0.21)	−0.50 (− 0.67 to − 0.31)	−0.27 (− 0.45 to − 0.09)	−0.30 (− 0.48 to − 012)	−0.43 (− 0.61 to − 0.24)	−0.90 (−1.07 to − 0.70)

## Discussion

This paper reports on the translation, adaptation and psychometric properties of the HLQ version of Asante-Twi (Local Ghanaian language) with a sample of 1234 caregivers with children under 5 years. This target group was selected because we needed the HLQ to assess the impact of a community malaria programme for children under 5 years on the health literacy levels of caregivers. The article highlights the importance of having reliable tools for assessing health literacy in African settings, which are very different from European and Western countries, and even Asian countries. This version of the HLQ for Ghana appears to have acceptable, if not quite perfect, psychometric properties, with dimension 9 (which is very ‘functional’) having a less well fit, and several other dimensions with borderline fits (< 0.80), and more items with factor loading < 0.60 than in the other translations. The interpretation of the findings from this study is discussed in the context of previous HLQ validation studies in other languages and in the context of the current study.

The results of the nine-factor CFA show that the model has an acceptable fit. However, in the one-factor analysis, the scale 9 ‘Understanding health information well enough to know what to do’ seemed to have high point estimates for RMSEA > 0.1 and low estimates for CFI and TLI ≤0.95, which means the model fits less well. Debussche et al. [15] reported a similar finding in the validation study of the French version of HLQ, where all scales had a good fit except scale 9. Furthermore, we observed that some question items of scale 9 loaded negatively on the scale although close to zero, a finding that stands out from other studies. This means the items negatively influence the scale or do not contribute to the construct, ‘understanding of health information well enough to know what to do’. The items included the questions how easy or difficult do you find it to: 1) accurately follow the instructions from healthcare providers? and 2) understand what health providers are asking you to do? Both questions reveal how people respond to instructions from health providers and should load well on understanding health information; this was, however, not the case in this context. This could be attributed to the translation and how the translated questions may have altered the intended meaning. As noted from the consensus meeting, many deliberations and discussions were assigned to these two questions, because the backward translations sounded stronger, exaggerating the intended meaning of the original version. However, the forward translations were approved as the panel agreed that they fitted the meaning of the original English version. Nonetheless, it is likely that the accepted forward translations might have had stronger meanings than the original version, and thus the exaggerations resulting from the translations might have shifted the focus from understanding to applying the information.

The less fitting model for scale 9 and the overall model could also be attributed to the context and cultural relevance of the construct. As this questionnaire measures health literacy as a multi-dimensional concept, with the dimensions constructed in a different context from Ghana, some of the items may not be factual [28] in Ghana and therefore will not support the model fit of the construct in Ghana. Thus, less fitting model does not always depict a bad model or bad dataset but the theory behind the concept, context differences and translation where necessary, could greatly influence how well the model fit. This is one reason why this study recommends further work on this questionnaire in Ghana, especially to re-examine scale 9 in the Ghanaian or similar contexts to improve on this measurement tool for such contexts [8, 12–14].

The difficulty levels of the scales appear to be higher than other validation studies except the Chinese [17], which was quite similar. However, the high difficulty levels in the Chinese study could be due to the sample consisting of older adults (60 years and above) [17]. This is supported by a similar finding by Bo et al., reporting that older adults are more likely to lack sufficient health literacy skills [3]. A high range of difficulty levels was primarily found in the second part of the questionnaire (scales 6 to 9), which is in line with findings from other validation studies on the HLQ [8, 12–14, 17]. However, the range of difficulty level for scales 6–9 from our findings is also higher (0.21–0.68) compared with the original English version (0.08–0.42) [8], but in line with the French version of the HLQ (0.32–0.69) [15]. The higher difficulty levels found for HLQ part 2 indicates a larger health literacy gap and suggests that most caregivers in this study need some form of support to empower them in their engagements with the health system for better health care and health outcomes.

Within HLQ Part 1, scale 1 ‘Feeling understood and supported by healthcare providers’ shows high difficulty levels for all of the four items ranging from 0.50–0.54. Comparatively, the difficulty levels for the items in this scale were higher than that recorded in the other validation studies [8, 12–15, 17] with the lowest levels ranging from 0.10–0.19 for the Australian version (original version) [8]. Considering the differences in development among the referenced countries, this result is expected from a relatively low resource setting such as Ghana.

The lack of health personnel in the Ghanaian health system may partly explain why as many as 60% of respondents report not having health provider support. Ghana has low provider to population ratios for both doctors (1 to 8481) and nurses(1 to 627) [29], thus, patients are likely not to get enough time with their healthcare providers. Furthermore, the low provider-patient ratios lead to pressure on the health workers that may negatively impact on their reliability and responsiveness [30]. This makes it difficult to have at least one healthcare provider to consistently support patients making health decisions. Thus, health provider support might not be a strong feature of the health system, and it is not surprising to see low scores in this study. In a better-resourced health system with good coverage and access, the scale ‘Feeling supported by healthcare providers’ would to a larger extent reveal individual competences in benefitting from the support from the health system compared with low resource settings, where access to such support is more limited. Thus, although the scale shows the ability of an individual to engage with health providers, it is likely also to reflect how the health system is responsive to the needs of the individuals depending on the setting. Any intervention to address low levels of health literacy in this dimension might differ between low and high resource settings, with focus on individual competences in the well-resourced system, as opposed to a focus on health system gaps and organisational responsiveness to health literacy in poorly resourced systems.

Almost 90% of the respondents in our study spoke a local language at home and only 10% spoke English at home. The difference in mean scores between these two groups in Table 4 showed that the English speakers scored higher in especially the last four scales, including navigating the health system. In this paragraph, we discuss language as a barrier [9, 10, 31] to navigating health systems especially when the official language is different from the local language(s). As English serves as the official language in Ghana, most written health information is in English, including labels on medications. This works well for English speakers at the detriment of most of the population. It is not surprising that for items 9.3 and 9.4 (read and understand written health information; read and understand medication labels) most respondents (68 and 64%, respectively; Table 2) had low scores, and thus found the task to be difficult. Such low scores were not evident in the other validation studies [12, 14, 15, 17], probably because their official language was predominantly the most spoken language in the country. Contrary to these low scores, the majority (> 70%) of respondents found it easy to understand and follow health information given orally (items 9.2 and 9.5).

These observations on language bring up discussions on the relevance of constructs developed in written cultures in oral cultures. The advantage of the HLQ is the possible oral administration which makes it useful even in Ghana which is predominantly an oral culture although it was developed in Australia, a written-cultural context. However, certain constructs may not fit as well in the Ghanaian setting as it did in Australia, other European settings and in China. In oral cultures, information, even when written, is often communicated orally to increase the understanding of the people of interest. Thus, for some constructs in the HLQ, despite the relevance of the constructs the items under the construct may not be applicable to the Ghanaian setting. For example, health workers would normally fill medical forms for patients after asking them the relevant details needed to fill the form and in addition, written health information and medication leaflets, are translated and communicated orally to patients. Therefore, although the construct of understanding health information is relevant, some items are quite abstract to the context. As demonstrated in the development of an item bank of health literacy questions in South Africa, tools for such settings should include both cognitive and factually based items to reflect the local context and increase the relevance and accuracy of the tool [28]. This emphasizes the importance of assessing construct validity in the translation process, not only to ensure that the constructs reflect their intents but most important to assess how constructs and items could be restated or transposed to suit the context without deviating from the concept of interest [32].

Discussions on language barriers to access and use of healthcare systems have led to changes in policies in western countries concerning adding interpreters or providing language courses for the target population who do not speak the official language [9, 10], e.g. immigrants and the Inuit population may not be good at speaking the official language of the country [10, 31]. Sometimes the ability of an individual to read and write does not necessarily mean that the person can comprehend the meaning of the words [33]. Although English is known by almost all groups across Ghana, it is not the day to day spoken language by most people and may thus hinder access to and effective use of the health system. Although healthcare providers speak the local languages, some messages might be lost in translation [10] and it becomes more problematic when the inscriptions on medications, medical forms and the information to navigate the health system are also in the official language. The challenge might be to provide written health information in all languages and dialects because of non-documentation of some of the dialects. Nevertheless, there is good reason to raise such discussions in countries facing similar language issues to find policies and approaches to curb this problem. The above also emphasises the importance of translating the questionnaire even in settings with English as the official language. Even if the questionnaire could be maintained in English, cultural and contextual adaptation might be necessary, e.g. perception of a health care provider and nuances of emphasis on certain meanings.

### Strengths and limitations

Although the HLQ is one of the recommended tools by WHO [11] in low and middle income countries, this is the first validation study on the HLQ in Africa and thus serves as a first-hand information on how this tool works in an African context. We translated the HLQ to the most commonly spoken Ghanaian language and validated it using a relatively large sample. The validated Twi version of the HLQ can now be considered for assessment of health literacy in Ghana and other neighbouring countries such as Benin and Côte D’Ivoire, which have sub-populations speaking Twi. This could also be useful in countries with a higher number of residents of Ghanaian origin to describe their health literacy profiles.

However, the interpretations of findings are limited to a special group of the Ghanaian population being caregivers with children under 5 years, because the HLQ was needed to assess the impact of a malaria programme for children under five on the health literacy levels of their caregivers. Caregivers, especially mothers, influence the health status of their families and are thus key people to target to improve health literacy. Our interest in this special group, however, limits the generalisability of the findings. Hence, we recommend testing the questionnaire in other population groups to improve its usefulness in the general population. Another limitation is the potential response bias, as a questionnaire on malaria preceded the HLQ. The sequence might have steered responses to reflect health literacy in managing malaria in children under five rather than managing general health as intended. It might be easier for a caregiver to agree to a statement like “I feel I have enough information to manage my health”, if the person has malaria in mind. This is because the high prevalence of malaria has led to much familiarity with health information on the disease [34]. However, we expect that a caregiver, who is for example confident in having sufficient information and in navigating the health system when reflecting on malaria, is in general likely to be more confident and be able to navigate the health system. Therefore, we believe that this potential bias is minor and would not likely alter the findings. Furthermore, malaria is the most common health condition accounting for 40% of all outpatient cases at health facilities [35]. Using it as a proxy for health literacy is thus appropriate.

In the data collection, although we received response from all contacted respondents, we skipped some households in the absence of the inhabitants. However, there is less likelihood for any bias because, data collection covered a period from morning to evening which met the presence of many, thus, not many households were skipped.

The use of a non-native English speaker is a non-standard translation method in reference to the translation integrity procedure adapted in this study. We acknowledge that this might have reduced the quality of the backward translation but not the entire translation process. Our process may have resulted in a backward translation with non-standardized lexical choices and a lingua franca translation influenced by the expressions of the native language of the translator. However, the consensus discussion with one of the authors of the questionnaire, who is a native English speaker brought out the shortcomings of the forward translations which were discussed and amended accordingly.

This study calls for further investigations on the validity testing of the HLQ in Ghana or a context with similar cultural characteristics to improve the construct and cultural relevance of the HLQ in such settings for to develop suitable health literacy responsive interventions.

## Conclusion

This study aimed at translating and assessing the psychometric properties of the HLQ in Twi, the most spoken Ghanaian local dialect. Running a confirmatory factor analysis, the nine-factor model seemed to have an acceptable fit and our finding suggests the need for validity testing and verification of the relevance of certain dimensions like scale 9 (understanding health information) in Ghana or contexts of similar settings especially in Africa. This is necessary for future health literacy responsiveness and for scaling-up of useful and context relevant interventions.

## Data Availability

The datasets used and analysed during the current study are available from the corresponding author upon reasonable request.

## Abbreviations

HLQ: Health Literacy Questionnaire
TIP: Translation integrity procedure
DWLS: Diagonal weighted least squares estimator
LAVAAN: Latent variable analysis
CFA: Confirmatory factor analysis
CFI: Comparative Fit Index
TLI: Tucker Lewis Index
RMSEA: Root Mean Square Error of Approximation
SRMR: Standard Root Mean Square Residual
CHPS: Community-based health planning and service
CHN: Community health nurse
ES: Effect size
